# Production, purification, sequencing and activity spectra of mutacins D-123.1 and F-59.1

**DOI:** 10.1186/1471-2180-11-69

**Published:** 2011-04-10

**Authors:** Guillaume G Nicolas, Gisèle LaPointe, Marc C Lavoie

**Affiliations:** 1 Département de Biochimie Microbiologie et Bioinformatique, Faculté des Sciences et Génie, Université Laval, Québec (Québec), G1K 7P4, Canada; 2 Centre de Recherche en Sciences et Technologie du Lait (STELA), Institut des Nutraceutiques et des Aliments Fonctionnels (INAF), Faculté des Sciences de l'Agriculture et de l'Alimentation, Université Laval, Québec (Québec), G1V 0A6, Canada; 3 Département de Stomatologie, Faculté de Médecine Dentaire, Université de Montréal, Montréal (Québec), H3C 3J7, Canada; 4 Department of Biological and Chemical Sciences, Faculty of Pure and Applied Sciences, The University of the West Indies, Cave Hill Campus, P.O. Box 64 Bridgetown, BB11000, Barbados

**Keywords:** bacteriocin, lantibiotic, mutacin, pediocin, *Streptococcus mutans*

## Abstract

**Background:**

The increase in bacterial resistance to antibiotics impels the development of new anti-bacterial substances. Mutacins (bacteriocins) are small antibacterial peptides produced by *Streptococcus mutans *showing activity against bacterial pathogens. The objective of the study was to produce and characterise additional mutacins in order to find new useful antibacterial substances.

**Results:**

Mutacin F-59.1 was produced in liquid media by *S. mutans *59.1 while production of mutacin D-123.1 by *S. mutans *123.1 was obtained in semi-solid media. Mutacins were purified by hydrophobic chromatography. The amino acid sequences of the mutacins were obtained by Edman degradation and their molecular mass was determined by mass spectrometry. Mutacin F-59.1 consists of 25 amino acids, containing the YGNGV consensus sequence of pediocin-like bacteriocins with a molecular mass calculated at 2719 Da. Mutacin D-123.1 has an identical molecular mass (2364 Da) with the same first 9 amino acids as mutacin I. Mutacins D-123.1 and F-59.1 have wide activity spectra inhibiting human and food-borne pathogens. The lantibiotic mutacin D-123.1 possesses a broader activity spectrum than mutacin F-59.1 against the bacterial strains tested.

**Conclusion:**

Mutacin F-59.1 is the first pediocin-like bacteriocin identified and characterised that is produced by *Streptococcus mutans*. Mutacin D-123.1 appears to be identical to mutacin I previously identified in different strains of *S. mutans*.

## Background

The excessive and often inappropriate use of antibiotics leads to a continuous increase and spread of antibiotic resistance among bacteria, thus making it imperative to discover and carefully use new antibacterial substances [1]. Bacteriocins are bacterial ribosomally synthesised proteinaceous substances with strong antibacterial activity, excellent structural stability, low immunogenicity, while resistance does not develop frequently [2-4]. One general mechanism of action of bacteriocins involves pore formation in target cells leading to the leakage of small molecules and cell death [4,5]. Bacteriocins from Gram positive bacteria can be grouped into three classes: class I which includes lantibiotics containing post-translationally modified amino acids such as lanthionine and dehydrated amino acids, class II non-lantibiotics, containing only common amino acids and class III containing bacteriocins with higher molecular mass (> 10 kDa) [2,4]. Lantibiotics (class I) are divided into type A (elongated linear peptides) and type B (globular peptides) [5]. Class II is subdivided into three subclasses, namely, class IIa (pediocin-like bacteriocins), class IIb (two-peptide bacteriocins) and class IIc (other one-peptide bacteriocins) [2]. The biosynthesis of bacteriocins requires the translation of the prepeptide, post-translational modifications such as dehydration and cyclisation reactions to form the thioether bridges in lantibiotics and cleavage of the leader peptide during transport outside of the cell [2,4]. *Streptococcus mutans*, a human indigenous oral bacterial species, is known to produce bacteriocins named mutacins [6]. It is believed that production of such mutacins may confer to *S. mutans *an advantage against competitive species living in the same niche [6]. To date, mutacins from class I and class II have been purified and characterised: the mono-peptide lantibiotic (mutacin B-Ny266), the di-peptide lantibiotic (mutacin GS-5), the mono-peptide non-lantibiotic (mutacin N) and the di-peptide non-lantibiotic (mutacin IV) [for review see reference 6 and references therein]. Production of more than one mutacin by a given strain has been experimentally demonstrated for several strains and is also predicted by bioinformatic analysis of sequenced strain genomes [6]. Mutacin-producing strains and some of their purified peptides have shown activity against Gram positive and some Gram negative bacteria *in vitro *and *in vivo *[7-9]. Because of their biochemical diversity and activity spectra, many applications can be expected for mutacins as antibiotics or food preservatives [3,10].

The main objective of our research is to further characterise mutacins to uncover new useful antibacterial substances active against bacterial pathogens. We previously classified 86 mutacin-producing strains into 24 groups (designated A to X) and subsequently seven clusters of activity were defined from the 24 type strains. This grouping was based only on their activity spectra towards other mutacinogenic strains and against various bacterial species including pathogens [8,11]. *S. mutans *59.1 and 123.1 were clearly distinct in their activity spectra and the mutacins produced by these strains were not genetically related to the well known lantibiotics (nisin, gallidermin, epidermin, subtilin) nor to previously well characterised mutacins (B-Ny266, B-JH1140 (mutacin III), J-T8 (mutacin II), H-29B) by using specific molecular probes [8,12]. We present here results on the production, purification and characterisation of mutacins F-59.1 and D-123.1.

## Results

Mutacin F-59.1 was produced in SWP and the activity was measured as 400 AU/mL while production of mutacin D-123.1 was achieved in semi-solid medium by using tryptic soy with yeast extract containing agarose. Activity of the crude mutacin D-123.1 preparation was measured to be 200 AU/mL.

**Figure 1:**
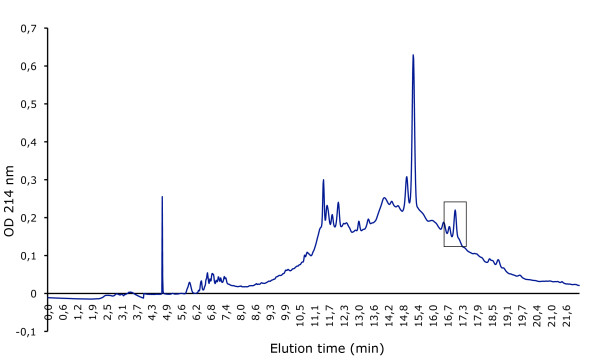
**Elution profile of mutacin F-59.1 on RP-HPLC**. Active peak is boxed.

**Figure 2:**
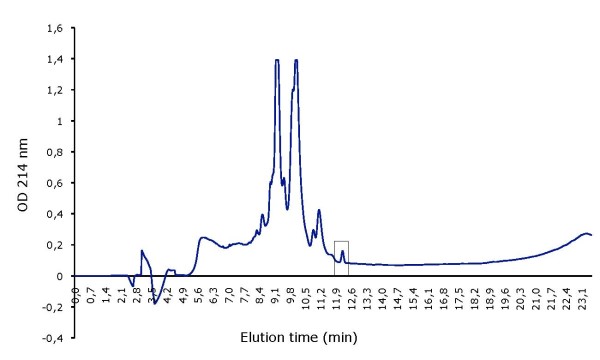
**Elution profile of mutacin D-123.1 on RP-HPLC**. Active peak is boxed.

Mutacins D-123.1 and F-59.1 were purified by successive steps of hydrophobic chromatography. Active fractions of mutacin F-59.1 purification were recovered with an elution gradient of 50%-60% methanol in 10 mM HCl (Figure 1) and those of mutacin D-123.1 with a 60%-70% gradient (Figure 2). The final specific activities were 3.2 × 10^5 ^AU/mg for the purified mutacin F-59.1 and of 1.6 × 10^5 ^AU/mg for the purified mutacin D-123.1 (Table 1).

**Table 1:** Purification of mutacins F-59.1 and D-123.1 by hydrophobic chromatography.

Step	Volume (mL)	Activity (AU/mL)	Total Protein (mg)	Total activity (AU.10^3^)	Specific activity (AU/mg)	Yield (%)	Purification (fold)
mutacin F-59.1							
Culture supernatant	1000	400	10000	400	40	100	1
Sep-Pak C_18_	95	3200	3000	304	101	76	2.5
C_18 _RP-HPLC	2	16000	0.1	32	3.2 × 10^5^	8	8 × 10^3^

mutacin D-123.1							
Culture supernatant	675	200	4320	135	31	100	1
Sep-Pak C_18_	50	1600	8	80	1 × 10^4^	59	320
C_18 _RP-HPLC	1	800	0.005	0.8	1.6 × 10^5^	0.2	5120

**Figure 3:**
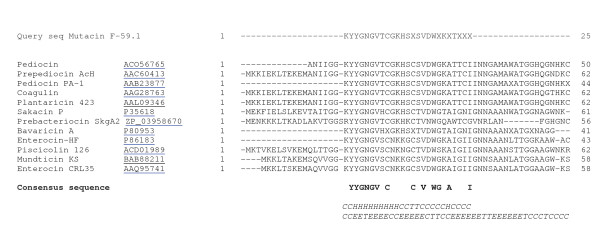
**Multiple sequence alignment of mutacin F-59.1 with homologous class IIa bacteriocins**. Consensus sequence appears in bold. Some of the leader sequences are shown with the double glycine motif. Underneath appears in italic the predicted secondary structure for mutacin F-59.1 and pediocin PA-1. Output classification is as follows: H, alpha-helix; E, extended strand; T, turn; C, the rest [43]. Accession numbers refer to bacteriocins in the protein database from the NCBI (AAC60413, [44]; AAB23877, [45]; AAG28763, [46]; AAL09346, [47]; P35618, [48]; P80953, [49]; ACD01989, [50]; BAB88211, [51]; AAQ95741, [52]).

**Figure 4:**
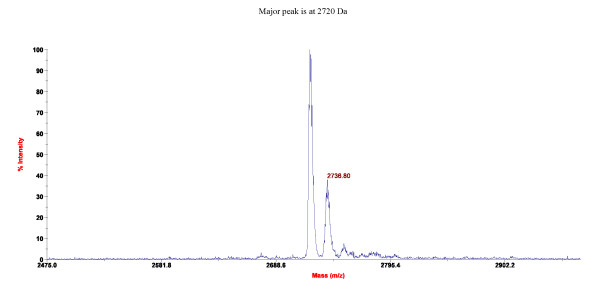
**MALDI-TOF-MS spectra obtained for pure mutacin F-59.1**.

A total of 25 amino acids were sequenced for mutacin F-59.1 and its identity with pediocin-like bacteriocins was confirmed by multiple alignment (Figure 3). The sequence revealed high levels of similarity to class IIa bacteriocins with the presence of the five residues of the common consensus sequence -YGNGV- and the two conserved cysteine residues at positions 9 and 14. The substitution of unidentified amino acids (annotated X) in the mutacin F-59.1 sequence with consensus amino acids found in our alignment (Figure 3) and those of others [2,13], revealed that the following N-terminal sequence KYYGNGVTCGKHS**C**SVDW**S**K**A**T**TNI **matches the molecular mass determined by MALDI-TOF MS analysis (2720 Da +/- 2 Da, due to the formation of the current disulfide bridge found between C9 and C14 in pediocin-like bacteriocins [2], (Figure 4)). The isoelectric point of mutacin F-59.1 (pI = 8.71) and secondary structure prediction with this sequence correlate well with other class IIa bacteriocins (Figure 3) [2,4].

**Figure 5:**
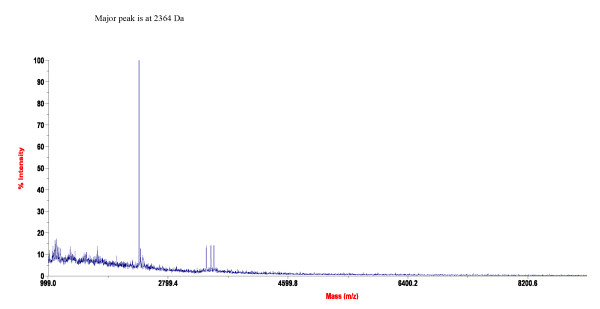
**MALDI-TOF-MS spectra obtained for pure mutacin D-123.1**.

The molecular mass for mutacin D-123.1 was computed to be 2364 Da (Figure 5). However, sequencing of the mutacin D-123 proved to be problematic. Edman degradation of native mutacin D-123.1 was blocked after the first residue (F). The sequence of only the first 9 amino acids was clearly obtained after the derivatisation procedure, but with at least two peaks at each cycle.

**Figure 6:**
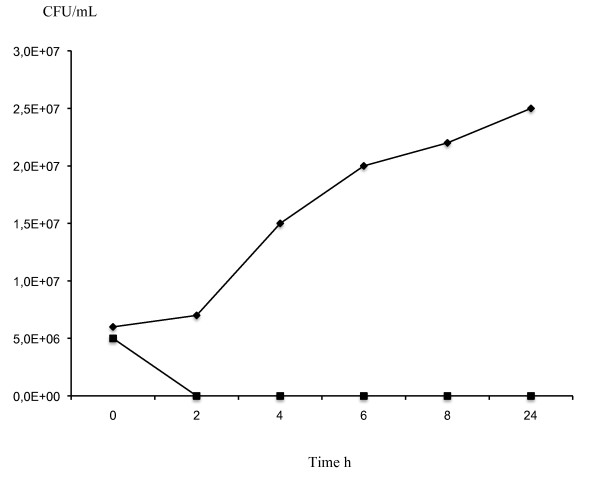
**Inhibitory action of purified mutacin F-59.1 against *Micrococcus luteus *ATCC 272**. Growth of cells was followed by measuring the viable count (CFU/mL) following the addition of purified mutacin F-59.1 (1600 AU/mL) (square line) or not for control (diamond line).

The growth of *M. luteus *ATCC 272 was inhibited immediately following the addition of a purified preparation of mutacin F-59.1 at 160 AU/mL as the viable count decreased rapidly and dropped to zero compared to the control. Over an incubation period of 24 h, the viable count of the test culture remained unchanged, suggesting that mutacin F-59.1 activity was bactericidal at the concentration tested (Figure 6).

The activity spectra observed for mutacins F-59.1 and D-123.1 show inhibition of a wide range of pathogenic bacteria including *Bacillus *spp., *Enterococcus *spp., *Listeria *spp.,

*Staphylococcus *spp. and *Streptococcus *spp. (Table 2).

**Table 2:** Inhibitory spectra of purified mutacins F-59

Indicator bacteria	Activity of mutacin (AU/mL)	
	
	D-123.1	F-59.1
*Bacillus cereus *ATCC 2	n.t.^a^	400
*Bacillus subtilis *ATCC 6051	n.t.	400
*Enterococcus faecium *ATCC 19434	0	1600
*Enterococcus faecalis *ATCC 27235	400	200
*Enterococcus hirae *ATCC 8043	200	200
*Lactobacillus salivarius *SMQ 876	n.t.	0
*Lactococcus lactis *ATCC 11454	400	400
*Listeria monocytogenes *ATCC 15313	400	200^b^
*L. monocytogenes *ATCC 700301 ScottA	200	200^b^
*L. monocytogenes *ATCC 700302 ScottA	200	200^b^
*L. monocytogenes *FRDC 1039	400	200^b^
*L. monocytogenes *FRDC 88571	400	200^b^
*Listeria murrayi *ATCC 25420	200	200^b^
*L. murrayi *HPB 30	400	200^b^
*Listeria ivanovii *HPB 28	400	200
*Listeria grayi *ATCC 19120	800	200
*Micrococcus luteus *ATCC 272	11600	3200
*Pediococcus acidilactici *UL5	400	800
*Staphylococcus aureus *ATCC 6538	n.t.	0
*S. aureus *ATCC 25923	0	0
*S. aureus *ATCC 43300	200	0
*S. aureus *R621	200	0
*Staphylococcus carnosus*	1600	800
*Streptococcus mutans *59.1	n.t.	200^b^
*S. mutans *123.1	200^d^	n.t.
*Streptococcus sobrinus *ATCC 27352	200	800
*Streptococcus salivarius *ATCC 25923	800	800
*Streptococcus pyogenes *ATCC 10389	200	0
*Streptococcus suis *serotype 2	400	0

ATCC (Manassas, VA, USA); HPB (Health Canada, Ottawa, ON, Canada); FRDC (Agriculture and Agrifood Canada, Sainte-Hyacinthe, QC, Canada).

^a^Not tested.

^b^Hazy inhibition zone was observed.

## Discussion

The inhibitory activity produced by the fermentation of *S. mutans *59.1 in SWP did not come from release of pediocin already present in the whey proteins or permeate used to make the medium because no inhibitory activity in SWP was detected from non-fermented nor purified medium against *M. luteus *ATCC 272 and also because many other *S. mutans *strains were unable to produce an inhibitory activity by fermentation of the same medium [14,15].

Of all the current microbiological broth media commonly used for the growth of *Streptococcus *sp., none permitted the production of a detectable level of mutacin activity by *S. mutans *123.1. Activity of mutacin D-123.1 was only detected after growth on solid medium. The production of some bacteriocins and mutacins is controlled by quorum sensing mechanisms which are better expressed when cells are grown at high density compared to lower cell density obtained in liquid culture [6]. For the isolation of mutacin D-123.1, agarose was preferred over agar as agar may contain compounds interfering with accurate detection of mutacin activity [16]. The nature of these compounds is still unknown, but divalent anions such as sulfates are suspected. As seen in previous work with other mutacins, purification yields were low (Table 1) and additional chromatographic steps will be necessary to improve yields and purity. The higher concentration of methanol used to recover mutacin D-123.1 suggests that the peptide is more hydrophobic than mutacin F-59.1. Collected samples of pure mutacin D-123.1 were very viscous because they probably retain part of the polymeric sugars from the agarose. However, with the methods used here, sufficient amounts of the substances were collected to carry out a preliminary characterisation of the peptides but the evaluation of their antibacterial spectrum was somewhat restricted.

The sequence of mutacin F-59.1 (25 residues) was shorter than the generally recognised size for pediocin-like bacteriocins which is between 37 and 48 residues [2,13]. This may be due to peptidase activity of the strain. Fifty three peptidases or peptidase homologues are found in the genome of *S. mutans *UA159 using the MEROPS database [17,18]http://merops.sanger.ac.uk. The pediocin-like bacteriocin sequence could thus be a substrate in its 25^th ^position for many of these peptidases. MALDI-TOF MS analysis revealed a major peak with an isotopic mass [M+H]^+ ^of 2720 Da for mutacin F-59.1 (Figure 4). This mass represents the lowest reported mass for an active naturally-produced pediocin-like bacteriocin after the study of Bhunia et al. [19]. The length of mutacin F-59.1 was sufficient to confer antimicrobial activity against several bacterial genera including *Bacillus *spp., *Enterococcus *spp., *Lactococcus *spp., *Micrococcus *spp., *Listeria *spp., and *Streptococcus *spp. (Table 2). Salvucci et al. [20] reported activity of short peptides derived from the NH_2_-terminus of enterocin CRL35 and other class IIa bacteriocins, suggesting that the C-terminus of pediocin-like bacteriocins is not essential for their inhibitory activity. Also, an active antimicrobial region in the NH_2_-terminus of this class of bacteriocin was identified by a bioinformatic approach [21]. The C-terminus section is known to confer specificity in the activity spectra of class IIa bacteriocins and to interact with their cognate immunity proteins [22]. Pediocin-like bacteriocins are unstructured in an aqueous solution and become structured when in contact with membrane-mimicking entities [2]. The electrostatic distribution along the molecule is highly polarized with most of the cationic residues concentrated in the N-terminal region. In the model class IIa bacteriocin sakacin P, the N-terminal residues in positions 7-9 (V-T-C) and 15-17 (S-V-D) are predicted to form an anti-parallel β-sheet-like structure stabilised by a conserved disulfide bridge, followed by a hairpin-like structure that consists of an amphiphilic α-helix in position 18-25 (W-G-K-A-I-G-I-I). A second α-helix normally found in pediocin-like bacteriocins at position 29-32 (S-A-A-N) with the C-terminal tail (residue 33 to the end) that folds back onto the central α-helix is absent in mutacin F-59.1. A flexible hinge is found in position 17 (D) between the N-terminal β strands and the hairpin-like C-terminal region [23]. Studies on the conformational changes of pediocin in an aqueous medium were conducted by Gaussier et al. [24]. The authors concluded that the flexibility of the protein ensures its activity and that the aggregation of the C-terminus caused a loss of activity. Lack of the C-terminus in mutacin F-59.1 should prevent the formation of such aggregates and does not disrupt the activity of the molecule. The predicted secondary structure of mutacin F-59.1 appears to differ slightly from that of pediocin PA-1. An α-helix is formed between residues 2 to 11 and a turn is found at position 14-15 as compared to position 18-19 of pediocin PA-1. The positions of the disulfide bridges were correctly predicted between positions C9-C14 for mutacin F-59.1 and between positions C9-C14 and C24-C44 for pediocin PA-1 (data not shown).

As for mutacin I, Edman degradation of native mutacin D-123.1 was blocked after the first residue (F), suggesting that the second residue (probably an S residue) was dehydrated as dehydrated amino acids in lantibiotics were shown to block Edman degradation [25,26]. Following close inspection using the relative intensity of each peak as a reference and the fact that ethanethiol treatment broke mutacin I into two fragments according to Qi et al. [25], therefore creating two N-termini peptides in the mixture to be sequenced, we reasoned and found the following partial amino acid sequence for mutacin D-123.1: F-SEC-SEC/DSER-L-SEC-L-SEC-SEC/DSER-L-(...)-P-SEC/DSER-F-N-SEC/DSER-Y-SEC-SEC. According to Meyer et al. [26], SEC results from the conversion of a dhA while a SEC signal accompanied by a DSER signal indicates residues involved in Lan (A) formation, making the thioether bridge. Based on these observations and by analogy to mutacin I, a more accurate, partial and truncated sequence with structural thioether bridges positions can be proposed for mature mutacin D-123.1. The sequence of the two separate fragments obtained for the mutacin D-123.1 is as follows:

Nter-F-S-S-L-S-L-C-S-L-(...)-P-S-F-N-S-Y-C-C

Nter-F-dhA-A-L-dhA-L-A-A-L-(...)-P-A-F-N-A-Y-A-A.

(A) residues are involved in Lan formation. At this stage, an accurate thioether bridge pattern of mutacin D-123.1 cannot be proposed unambiguously. The mass of mutacin D-123.1 matched exactly that calculated for the lantibiotic mutacin I produced by *S. mutans *CH43 and UA140 (2364 Da) [25,27]. This observation strengthens the apparent identity between mutacin D-123.1 and mutacin I.

The activity spectra of purified mutacins F-59.1 and D-123.1 are in accordance with the antibacterial activity spectra of the respective producing strains inhibiting *Bacillus cereus*, *Enterococcus *spp., *Listeria monocytogenes*, *Staphylococcus *spp. and *Streptococcus *spp. using the deferred antagonism assay and thus observed for other purified pediocin-like bacteriocins and mutacins [2,7,8,13,19,22,27]. However, some of the strains tested, particularly *Listeria *spp., were less sensitive to the activity of purified mutacin F-59.1 than to the producer strain itself [8]. This may be due to the production by *S. mutans *59.1 of more than one mutacin in solid medium having activity against *Listeria *spp.. Also, resistance to pediocin-like bacteriocins in *Listeria *species has already been reported and can be physiologically or genetically acquired [28,29]. Low levels of resistance are caused by alterations in membrane lipid composition while high resistance levels involved the loss of a mannose permease component [30,31].

Nisin resistance is also reported and is related to membrane composition [32] or alterations in the cell wall [33]. Our results show that nisin-resistant *Listeria *strains were still sensitive to the lantibiotic mutacin D-123.1. Lipid II-targeted lantibiotics that are too short to form a pore across the bilayer membrane can still maintain their antibacterial activity to be able to kill the nisin-resistant strains In a similar manner, mutacin D-123.1 could act by trapping lipid II from the septum, blocking peptidoglycan synthesis and leading to cell death [34]. Moreover, activity of mutacin D-123.1 against antibiotic-resistant *Enterococcus *spp. and *Staphylococcus *spp. stresses its potential as a new antibiotic. Weak activity of mutacins F-59.1 and D-123.1 were observed against their respective producing strains (*S. mutans *59.1 and 123.1) as compared to the highly sensitive strain *M. luteus *ATCC 272, which suggests that the respective strains are able to produce specific self-immunity factors. Bacteriocin biosynthesis genes are generally co-transcribed with a gene encoding a cognate immunity protein ensuring protection of the producing cell against the lethal activity of the bacteriocin they produce [4].

Pediocin-like bacteriocins were identified in a wide variety of Gram positive bacteria such as *Bacillus *spp., *Carnobacterium *spp., *Enterococcus *spp., *Lactobacillus *spp., *Leuconostoc *spp., *Listeria *spp. [2,13]. While high heterogeneity has been observed in the genetic determinants coding for production of mutacins [12,35], this is the first report of a pediocin-like mutacin produced by *S. mutans*, which further extends the distribution of pediocin-encoding genes as well as the antibacterial spectra of *S. mutans *against pathogens sensitive to class IIa bacteriocins. From the two genomes of *S. mutans *strains available in public databases (UA159 in GenBank under accession number AE014133 and NN2025 in the DNA databank of Japan under accession number AP010155) [17,36], no lantibiotic nor pediocin-like bacteriocin sequences were found, although many bacteriocin-related genes are detected [6]. Only some entries with bacteriocin_II superfamily proteins (pfam01721) in the NCBI database matched two pediocin family proteins from *Streptococcus bovis *ATCC 700338 and *Streptococcus mitis *ATCC 6249 (EFM26697.1 and EFM30880.1). To our knowledge, only a *Streptococcus uberis *strain was shown to produce a pediocin-like bacteriocin named ubericin A [37]. Production of bacteriocins is widely distributed among strains of *S. mutans*. Lantibiotic-type mutacin production is sporadically detected from strains isolated from different origins; this strongly suggests the existence of a common genomic ancestor element for lantibiotic biosynthesis [6]. Comparative genomic analysis reported that dispensable genes exist and have been scattered through horizontal genetic transfer in various *S. mutans *strains. These optional mobile genes may be selected when they provide competitiveness to the strains as in the case of bacteriocin production to compete with the numerous other bacterial species resident in the oral cavity [38].

## Conclusion

Two bacteriocins from *S. mutans *have been isolated and characterised in terms of molecular mass, sequence and activity spectra. Mutacin F-59.1 is related to pediocin-like bacteriocins and is the first one shown to be produced by *S. mutans*. Mutacin D-123.1 appears identical to mutacin I in molecular mass and in the N-terminus sequence. Antibacterial activity spectra of these mutacins indicate promising potential application by inhibiting numerous bacterial pathogens. More research remains to be done to increase the low yields of mutacin production and purification.

## Methods

### Bacterial strains and media

*Streptococcus mutans *59.1 and 123.1 produce mutacins F-59.1 and D-123.1 respectively [8]. *Micrococcus luteus *ATCC 272 (ATCC, Manassas, VA, USA) was used as the indicator strain for the mutacin activity assays. All bacteria were routinely grown aerobically at 37°C in TSBYE made of TSB (Difco laboratories, Detroit, MI, USA) supplemented with 0.3% yeast extract (Becton Dickinson & Co., Cockeysville, MD, USA) or on TSAYE plates made of TSA (Difco) enriched with 0.3% yeast extract. *Lactobacillus salivarius *strain (provided by Sylvain Moineau, Université Laval, Québec, QC, Canada) was cultivated aerobically at 30°C in MRS medium (Oxoid, Nepean, ON, Canada). Other bacterial strains used for the inhibitory spectra determination are described in Mota-Meira et al. [7] and Morency et al. [8]. *Staphylococcus carnosus *was obtained from the strain collection of the Department of Microbiology, Biochemistry and Bioinformatics (Université Laval).

### Production of mutacins

An overnight culture of the producing strain *Streptococcus mutans *59.1 in TSBYE was used to inoculate (1% v/v) 2 L of supplemented whey permeate (SWP) consisting of cheese whey permeate 6% (w/v) (kind gift from Agropur Coop., Granby, QC, Canada) supplemented with 1% CaCO_3 _(Anachemia, Montréal, QC, Canada) and 2% yeast extract (Institut Rosell, Montréal, QC, Canada). The culture was incubated 48 h at 37°C under aerobic conditions, centrifuged at 10 000 × *g *for 10 min and the supernatant was heated at 70°C for 10 min to destroy the remaining cells and enzyme activity [14].

Mutacin D-123.1 was produced in TSBYE (Difco) containing 0.5% agarose (Difco). Batches of this medium (4 L) were stab inoculated with a culture of *S. mutans *123.1 grown in TSBYE and incubated for 72 h at 37°C. After growth, the culture was scraped, aliquoted into centrifuge bottles and frozen overnight at -20°C. The bottles were then centrifuged at 4000 × *g *for 60 min and 8000 × *g *for 30 min at room temperature. The resulting supernatant was filtered through glass fibers and Whatman no. 1 filter paper to remove agarose fines then stored at 4°C.

### Purification of mutacins

Purification of the two mutacins was achieved by two hydrophobic chromatography steps as previously described [15,39] by replacing TFA with HCl (10 mM) [40]. Briefly, the active preparation was loaded on a Sep-Pak^® ^Vac 35 cc (10 g) t-C_18 _Cartridge (Waters Corporation, Milford, MA, USA). Cartridges were first equilibrated with 500 mL of methanol followed by 500 mL of deionized distilled water. Antibacterial compounds were eluted with successive steps of 500 mL of water:methanol mixtures increasing the gradient of methanol by 10% from 0 to 100% in 10 mM HCl. This was carried out at a flow rate of 1 mL/min and UV detection at 214 nm. The final purification step was carried out by reverse phase chromatography (RP)-HPLC analysis (Beckman Gold Model, Coulter Canada Inc., Mississauga, ON, Canada) using an analytical C_18 _column (Luna 5 μ C18(2), 250 × 4.6 mm, 4 × 3.0 mm, Phenomenex, Torrance, CA, USA). Elution was carried out with solvent A (5% acetonitrile, 10 mM HCl) and solvent B (60% acetonitrile, 10 mM HCl) and recorded at 214 nm. The following program of elution was developed: 0 to 3 min, constant 100% A; 3 to 15 min, a linear gradient from 100% A to 100% B; 15 to 20 min, constant 100% B; 20 to 23 min, a linear gradient from 100% B to 100% A. A flow rate of 1 mL/min was used. The column was maintained at 39°C with a column heater. Active fractions were manually collected, subsequently dried in a Speed-Vac^® ^concentrator (Model SC110A, Savant Instrument Inc. Farmingdale, NY, USA) and then kept at -20°C until processing. Protein concentration in active fractions was determined using the BioRad DC protein assay (BioRad, Mississauga, ON, Canada).

### Activity assay of mutacins

Mutacin activity was determined by the spot test using *Micrococcus luteus *ATCC 272 as sensitive strain where two-fold dilutions were prepared in acidified (pH 2) peptone water (0.5%) [14]. Antibacterial activity spectra of purified mutacins was tested against a panel of bacterial strains using the critical dilution method combined with the spot test method as described previously [14]. Briefly, overnight cultures of test strains in TSBYE were diluted in fresh broth before inoculating 5 mL of soft agar (0.75%) in order to obtain confluent lawns of growth on TSAYE plates. Five μL of purified mutacins, diluted in acidified (10 mM HCl) distilled water to promote solubility of the peptides, were deposited on the lawn and allowed to dry before appropriate incubation. Mutacin activity was expressed in AU/mL and corresponds to the reciprocal of the highest dilution showing a noticeable inhibition zone on the lawn [14].

### Amino acid sequencing procedure

Alkaline ethanethiol derivatisation as described by Meyer et al. [26] was performed prior to sequencing of mutacins. Briefly, the purified sample was vacuum dried and was dissolved in 30 μL of a derivatisation mixture composed of 1.4 M ethanethiol and 0.5 M NaOH in 46% aqueous ethanol. The sample was then incubated for 60 min at 50°C in limited oxygen atmosphere. The reaction was stopped by the addition of 2 μL of glacial acetic acid (Sigma-Aldrich, St Louis, MO, USA) just before sequencing. Pure mutacin B-Ny266 was used as control for the Edman degradation with the alkaline ethanethiol derivatisation procedure [39]. Automated Edman degradation was performed on a protein sequencer (ABI Procise cLC, Applied Biosystems, Foster City, CA, USA) at the Biotechnology Research Institute (Montréal, QC, Canada). Amino acids were identified by capillary HPLC on a C_18 _0.8 × 150 mm column.

### Characterisation of mutacins by bioinformatic analyses

Homology searches were carried out with the National Center of Biotechnology Information (NCBI) using the basic local alignment search tool for protein (BLAST-P) with default parameters [41]. The constraint-based multiple alignment tool (COBALT) from NCBI was used with the default parameters to perform alignment. The primary and secondary structures of the mutacin F-59.1 were analyzed by the ExPASy Proteomics Server http://ca.expasy.org/tools/#proteome[42] and the SCRATCH protein predictor http://scratch.proteomics.ics.uci.edu/[43].

### Molecular mass analysis

The molecular masses of mutacins (D-123.1 and F-59.1) were determined from pure HPLC fractions by MALDI-TOF MS analyses at the Mass Spectrometry Laboratory of Molecular Medicine Research Centre (University of Toronto, Toronto, ON, Canada). A saturated β-cyano-4-hydroxycinnamic acid in 70% acetonitrile and 0.1% TFA was used as the matrix solution. One μL of peptide sample was spotted on the sample target, and then 1 μL of saturated matrix solution was added on the top. After the crystal was formed, the sample target was inserted into the mass spectrometer. MALDI MS was acquired in linear mode at positive on Applied Biosystems Voyager-DE STR MALDI-TOF mass spectrometer (Applied Biosystems, Foster City, CA, USA) equipped with a 337 nm laser. Acceleration voltage was set at 20 kV, grid voltage at 94%, guide wire at 0.05%, and delay time at 175 nsec. The mass spectra were externally calibrated by the molecular weights of a mixture of standard peptides. The mass accuracy is typically 0.05%.

### Protein sequence accession number

The protein sequence data of the mutacin F-59.1 appears in the UniProt Knowledgebase under the accession number P86386.

### Inhibitory effect of mutacin F-59.1

One milliliter of active preparation (1600 AU/mL) adjusted to pH 7.0 was filter sterilised then added to 10 mL of an early-log-phase culture of *Micrococcus luteus *ATCC 272 grown in TSBYE. Bacterial culture in TSBYE was used as a negative control. The viable count in CFU/mL was determined at intervals for up to 24 h for samples and control during incubation at 37°C by plating 100 μL of an appropriate dilution in peptone water (0.1%) on TSAYE incubated at 37°C at least 24 h.

## Abbreviations

ATCC: American Type Culture Collection; AU: arbitrary units; CFU: colony-forming units; dhA: 2,3-didehydroalanine; DSER: PTH-dithiothreitiol adduct of dhA; FRDC: Food Research and Development Center; Lan: lanthionine; HCl: hydrochloric acid; HPB: Health Protection Branch; MALDI-TOF MS: matrix assisted laser desorption ionisation-time of flight mass spectrometry; MRS: de Man Rogosa Sharpe; nsec: nanosecond; PTH: phenylthiohydantoin; RP-HPLC: reverse-phase high-pressure liquid chromatography; SWP: supplemented whey permeate; SEC: S-ethylcysteine; TFA: trifluoroacetic acid; TSA(B)YE: trypticase soy agar (broth) yeast extract; Amino acid one letter code is used.

## Authors' contributions

GN participated in project conception, coordinated and carried out most of the experiments, analysed and interpreted data and wrote the manuscript. GL designed and supervised the analyses and corrected the manuscript. MCL conceived the study and participated in its design as well as in correction of the manuscript. All authors read and approved the final manuscript.
